# LKB1 biology: assessing the therapeutic relevancy of LKB1 inhibitors

**DOI:** 10.1186/s12964-024-01689-5

**Published:** 2024-06-06

**Authors:** Charles B. Trelford, Trevor G. Shepherd

**Affiliations:** 1 The Mary &, John Knight Translational Ovarian Cancer Research Unit, London Regional Cancer Program, 790 Commissioners Road East, Room A4‑921, London, ON N6A 4L6 Canada; 2 Department of Anatomy and Cell Biology, Schulich School of Medicine and Dentistry, Western University, London, ON Canada; 3 Department of Oncology, Schulich School of Medicine and Dentistry, Western University, London, ON Canada; 4 Department of Obstetrics and Gynaecology, Schulich School of Medicine and Dentistry, Western University, London, ON Canada

**Keywords:** Tumour Suppressor, Liver Kinase B1 (LKB1), STE-20-related Kinase Adaptor Protein (STRAD), Mouse Protein 25 (MO25), Adenosine Monophosphate-Activated Protein Kinase (AMPK), Adenosine Monophosphate-Activated Protein Kinase-related Kinases (ARKs), Autophosphorylation, Post-translational Modifications, LKB1 Inhibitors, Drug Discovery

## Abstract

Liver Kinase B1 (LKB1), encoded by Serine-Threonine Kinase 11 (*STK11*), is a master kinase that regulates cell migration, polarity, proliferation, and metabolism through downstream adenosine monophosphate-activated protein kinase (AMPK) and AMPK-related kinase signalling. Since genetic screens identified *STK11* mutations in Peutz-Jeghers Syndrome, *STK11* mutants have been implicated in tumourigenesis labelling it as a tumour suppressor. In support of this, several compounds reduce tumour burden through upregulating LKB1 signalling, and LKB1-AMPK agonists are cytotoxic to tumour cells. However, in certain contexts, its role in cancer is paradoxical as LKB1 promotes tumour cell survival by mediating resistance against metabolic and oxidative stressors. LKB1 deficiency has also enhanced the selectivity and cytotoxicity of several cancer therapies. Taken together, there is a need to develop LKB1-specific pharmacological compounds, but prior to developing LKB1 inhibitors, further work is needed to understand LKB1 activity and regulation. However, investigating LKB1 activity is strenuous as cell/tissue type, mutations to the LKB1 signalling pathway, STE-20-related kinase adaptor protein (STRAD) binding, Mouse protein 25-STRAD binding, splicing variants, nucleocytoplasmic shuttling, post-translational modifications, and kinase conformation impact the functional status of LKB1. For these reasons, guidelines to standardize experimental strategies to study LKB1 activity, associate proteins, spliced isoforms, post-translational modifications, and regulation are of upmost importance to the development of LKB1-specific therapies. Therefore, to assess the therapeutic relevancy of LKB1 inhibitors, this review summarizes the importance of LKB1 in cell physiology, highlights contributors to LKB1 activation, and outlines the benefits and risks associated with targeting LKB1.

## Introduction

Liver kinase B1 (LKB1), encoded by serine-threonine kinase 11 (*STK11*), is a master serine-threonine kinase important for cell polarity, migration, and metabolism during fetal development throughout adulthood [1]. For this reason, *STK11* genomic instabilities are embryonic lethal [2, 3] and linked to pathologies including Peutz-Jeghers Syndrome and tumourigenesis [4]. Although the inactivation of LKB1 is associated with spontaneous tumour formation in many cancers [5–7], recent evidence suggests that LKB1 deficiency impairs invasion and metastasis in some cancer types [8, 9]. Therefore, there are concerns with labelling *STK11* as a tumour suppressor which has fostered emerging strategies utilizing LKB1 antagonists for disease therapy. Prior to generating LKB1 inhibitors, investigators must consider the therapeutic potential and risk factors associated with LKB1 ablation balancing the role of LKB1 in pathology against potential consequences of inactivation. Given that LKB1 activity is dependent on cell/tissue type, development, expression, splicing, protein–protein interactions, post-translational modifications, and cellular localization, this review seeks to summarize the role of LKB1 in cell biology with an in-depth analysis of factors essential to LKB1 stability and activity. Using this information, the therapeutic relevancy of LKB1 inhibitors will become apparent.

## LKB1 expression and structure

Since the discovery of *STK11*, identifying the promoter, regulatory elements, and transcription factors of interest were made possible through developing online databanks for conserved genetic structures and computational algorithms that assign function based on genetic patterns and sequence homology. The human genome browser of the Encyclopedia of DNA Elements identified a previously described DNaseI hypersensitivity region flanking the 5’ end of *STK11*. Sequencing this genomic region identified cis-regulatory elements including consensus sequences for CCAAT boxes and FOXO transcription factors [10]. Another group identified a p53 binding site within the promoter and verified that *TP53* overexpression upregulated *STK11* expression and protein levels [11].

Reverse transcription of the 1302 bp *STK11* cDNA sequence revealed the exon–intron structure of LKB1 [4]. Spanning 23 kbp on chromosome 19p human *STK11* consists of ten exons where only exons 1–9 are transcribed [1]. Mature LKB1 enzymes consist of a serine-threonine kinase domain (aa 44–309) flanked by N-terminal (aa 1–43) and C-terminal (aa 310–433) regions. The C-terminal region contains motifs commonly targeted for post-translational modifications including phosphorylation, acetylation, S-Nitrosylation, and farnesylation [12, 13]. Alternatively, mutating the N-terminal PRRKRA (aa 38–43) motif blocked LKB1 nuclear shuttling [14] suggesting that it functions as a single basic type nuclear localization signal [15]. Although LKB1 is ubiquitously expressed, many cells and tissues produce 48 kDa short (404 aa) and 50 kDa long (433 aa) LKB1 isoforms that differ in exon 9 splicing [16]. Most tissues express long LKB1 isoforms whereas some tissues, such as the testis, preferentially express the short isoform [17, 18]. Given that short isoforms replace 63 C-terminal residues with 39 unique residues [19], motifs subject to post-translational modifications in long LKB1 isoforms are absent. Furthermore, short LKB1 isoforms contain a serine 399 phosphorylation site in the C-terminal region responsible for dictating the enzymes cellular localization [19]. Taken together, splicing alters LKB1 activity [20, 21], which is observed when male mice lacking short LKB1 isoforms were sterile regardless of the expression of LKB1 long isoforms [19, 22].

The N-terminal nuclear localization signal recognized by importin-ɑ mediates the nuclear translocation of LKB1 [23] but active LKB1 predominately resides in the cytoplasm [24]. The loss of cytoplasmic retention is often observed in LKB1 mutants [25] and despite some LKB1 mutants localizing to the cytoplasm, kinase assays indicate that these mutants are catalytically inactive [26]. Several missense mutations to the LKB1 kinase domain implicated in Peutz-Jeghers Syndrome including W308C, L67P, L182P, G242V, and R297S mutations reduce enzymatic activity by distorting LKB1 structure [4]. Using automated comparative protein model tools, it is understood that W308C mutants impair enzyme activity by forming a disulfide bridge with C158 [27]. Alternatively, L67P, L182P, G242V, and R297S mutants disrupt secondary β-sheet formations, which alters protein folding and activation loop conformation [28]. Therefore, LKB1 activity is dependent on the conformational state of LKB1, which regulates the accessibility of the nuclear localization signal, kinase domain, and C-terminal regions subject to post-translational modifications [29].

## Characterizing the heterotrimeric LKB1-STRAD-MO25 complex

Regardless of its broad expression patterns, kinase and cellular localization assays indicate that monomeric LKB1 activity is low [30] and primarily localizes to the nucleus [24], respectively. Baas et al., 2003, revealed that unlike most kinases that are activated via phosphorylation, LKB1 activity is upregulated allosterically through protein–protein interactions [29, 31]. LKB1 binding to STE-20-related kinase adaptor protein (STRAD) and Mouse protein 25 (MO25) triggers nucleocytoplasmic shuttling and a conformational change in which the LKB1 activation loops lacks phosphorylation yet displays increased enzymatic activity [32]. Prior to targeting LKB1 activity, characterizing how STRAD and MO25 activate LKB1 and each of their respective roles in the heterotrimeric complex is of upmost importance.

### STE-20-related kinase adaptor protein (STRAD) is a allosteric activator of LKB1

A yeast two hybrid screen of a fetal brain library and co-immunoprecipitation assays identified STRAD as a potential binding partner for LKB1 [31]. Given that sequencing STRAD suggested that it lacked residues indispensable for kinase activity [33], and a radioactive kinase assay demonstrated no STRAD kinase activity, it was labeled a pseudokinase [31]. The STRAD pseudokinase domain binds to the LKB1 kinase domain and enhances its catalytic activity. Despite having little effect on LKB1 substrate specificity, STRAD enhances LKB1 autophosphorylation at T185, T336, T363, and T402 as well as substrate phosphorylation [31].

I*n vitro* radioactive kinase assays verified that SL26, a 9 bp in frame deletion at the C-terminus of LKB1, retains intrinsic kinase activity but were unable to form complexes with STRAD. Further analysis found that SL26 accumulated in the nucleus and displayed reduced cytoplasmic retention [25]. Transcriptome analysis of HeLa cells transfected with wild-type *STK11* or SL26 revealed that despite retaining catalytic activity in vitro, genes targeted by wild-type LKB1, such as the wnt/β-catenin pathway, remain unchanged in the presence of SL26 [34]. Additionally, both LKB1 and SL26 bind to the helicase domain of brahma-related gene 1 using the N-terminal region but only wild-type LKB1-mediated brahma-related gene 1-dependent growth arrest [35]. Therefore, STRAD binding is essential for the nucleocytoplasmic shuttling of LKB1 and functions including targeting transcription, brahma-related gene 1 growth suppression, and many other processes reviewed elsewhere [30, 36].

**Fig. 1:**
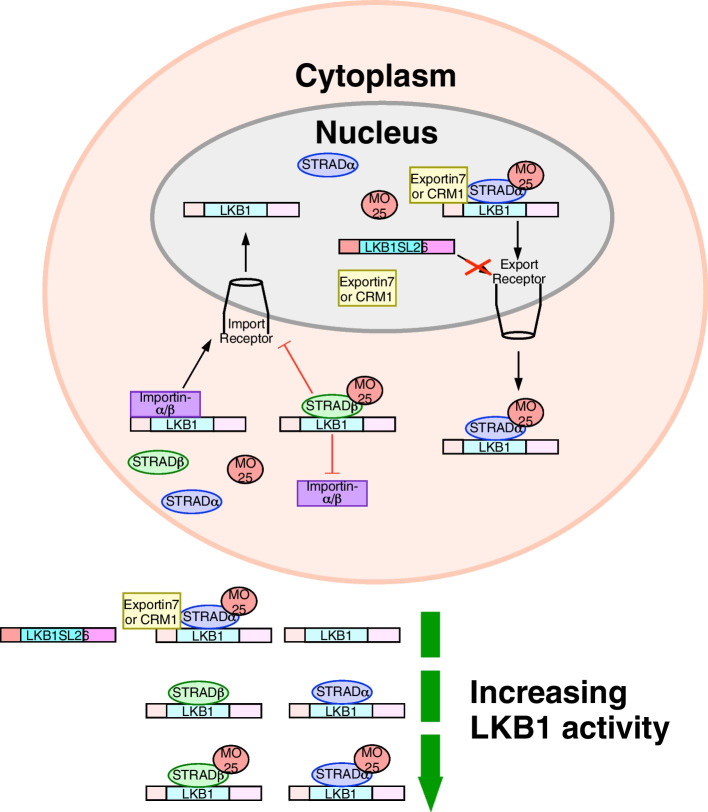
The effect of STRAD and MO25 on LKB1 nucleocytoplasmic shuttling and function Catalytically active LKB1 resides in the cytoplasm but is stored in the nucleus where it has little activity. The nuclear localization signal within LKB1 binds to importinɑ/β, which allows LKB1 to cross nuclear import receptors. STRADɑ/β binds to the kinase domain increasing the cytoplasmic retention of LKB1. STRADβ prevents binding to importin ɑ/β in the cytoplasm whereas STRADɑ binds to nuclear export receptors, such as exportin7 and CRM1, which allows LKB1 to cross nuclear export receptors. LKB1 activity is greatest when mouse protein 25 (MO25) binds STRAD and STRAD is bound to LKB1. LKB1 SL26 mutants have little enzymatic activity because they are unable to bind STRAD leading to the nuclear retention of LKB1 SL26

Humans express two different isoforms of STRAD (STRADɑ and STRADβ) that share functional similarities. In fact, STRADɑ and STRADβ are redundant in axonogenesis and cell survival in the developing cell cortex [37]. Each STRAD isoform co-precipitates with LKB1, induces its autophosphorylation, and promotes cytoplasmic localization [24]. Surprisingly, due to variations in the N-terminal and C-terminal sequences responsible for interacting with nuclear export signals, only STRADɑ efficiently interacts with nuclear export receptors, such as exportin7 and CRM1, to stimulate LKB1 nucleocytoplasmic shuttling. Alternatively, in vitro binding assays suggest that STRADβ disrupts LKB1-importin-ɑ interactions [23]. Therefore, through different mechanisms both STRAD isoforms increase cytoplasmic localization of LKB1 (Fig. 1).

Despite sharing functional similarities, only *STRADA* knockout decreased LKB1 protein levels in the embryonic cerebral cortex of mice [37]. Alternatively, STRADβ, but not STRADɑ, is essential for LKB1 localization to cilia [38]. Although other variations in their activity have been reported, more work is necessary to discern functional differences between STRAD isoforms that may contribute to pathologies driven by LKB1 activity. Furthermore, STRAD and LKB1 may have mutually exclusive roles as STRADɑ regulates neuronal polarity and synapse organization in *Caenorhabditis elegans* through an LKB1-independent mechanism [39]. These results were repeated in LKB1-null human lung cancer cell lines where STRADɑ forms a complex with p21-activated protein kinase 1 to regulate polarity and facilitate cell invasion [40].

### Mouse Protein 25 (MO25) enhances STRAD activity

Small interfering RNA (siRNA) targeting of *MO25* reduced the amount of STRAD immunoprecipitated with LKB1 [24], which suggested that MO25 stabilizes the LKB1-STRAD complex [32]. Although the co-expression of LKB1 and MO25 did not impact LKB1 localization, the cytoplasmic shift in LKB1 was greatest during MO25 and STRAD co-expression [24]. Further analysis found that MO25 is indirectly associated with LKB1 through STRAD binding in which ATP and MO25 maintain STRAD in an active conformation [41]. Mutating each STRAD residue to alanine indicated that the last three residues of STRAD (Trp-Glu-Phe) mediate MO25 binding. Together, LKB1-STRAD-MO25 function as a heterotrimer where MO25 and STRAD modulate LKB1 nucleocytoplasmic shuttling and conformational state (Fig. 1).

Like STRAD, MO25 has two isoforms expressed in humans (MO25ɑ and MO25β) that both co-precipitate with LKB1 [32]. There are numerous LKB1-independent MO25 functions as several STE-20 family kinases have been reported to bind to MO25. For instance, MO25 binding to STE-20/SPS-1-related proline-alanine-rich protein kinase and oxidative stress response kinase increases their ability to phosphorylate numerous ion co-transporters [42]. These results have been reproduced in *Drosophila* distal tubules as MO25 maintains transepithelial ion transport through With No Lysine Kinase signalling [43]. Given that only *MO25A*^−/−^ and *MO25B*^−/−^ double knockout mice develop the Gitelman-like phenotype characterized by hypokalemic alkalosis, hypomagnesemia and hypocalciuria, there is significant overlap in MO25ɑ and MO25β activities [44]. Despite differences in isoform activity being relatively unknown, MO25 regulates salt transport, distal tubule mass, and blood pressure in a LKB1-independent manner.

## Investigating LKB1-STRAD-MO25 activity

Despite both STRAD and MO25 having LKB1-independent functions, most investigations highlight their impact on cell biology through forming a 1:1:1 heterotrimeric complex with LKB1. The LKB1-STRAD-MO25 complex is a master regulator of cell polarity [45], metabolism [46], stress responses [47], migration [9], proliferation [48], and several other processes important to energy expenditure and homeostasis. LKB1 modulates these processes through downstream Adenosine Monophosphate-Activated Protein Kinase (AMPK) [49] and AMPK-related Kinases (ARKs) [50].

### The function of the LKB1-AMPK signalling *axis*

AMPK was the first LKB1 target identified as LKB1 significantly increased bacterial AMPK T172 phosphorylation, which was also observed in a similar study using rat AMPK [49, 51]. AMPK functions as a serine-threonine kinase that forms a heterotrimeric complex consisting of a catalytic subunit, AMPKɑ, and two regulatory subunits, AMPKβ and AMPKγ [52]. AMPK functions as an energy sensor that detects fluctuations in the AMP:ATP ratio using four cystathionine-β-synthase domains within AMPKγ [53]. In energy rich conditions, ATP binds to the cystathionine-β-synthase and allosterically deactivates AMPK. Alternatively, ATP depletion during nutrient deprivation increases AMP binding to Bateman domains located between the cystathionine-β-synthase domains. AMP binding allosterically activates AMPK and protects AMPK from dephosphorylation [54, 55]. Ultimately, AMP binding initiates a conformational rearrangement of AMPKɑ in which the catalytic T172 within the T-loop of the kinase domain becomes accessible to upstream AMPK kinases [56]. Although most investigations of AMPK function and regulation have focused on AMPKɑ and AMPKγ subunits, AMPKβ is necessary for interactions involving carbohydrates [57], and is post-translationally modified to upregulate or downregulate the enzymatic activity of AMPKɑ [58]. Currently, there is no evidence linking LKB1 complex assembly to AMP levels, yet AMP stimulated LKB1-dependent AMPK T172 phosphorylation in rat liver [54]. Although this finding is controversial, evidence that constitutively active LKB1 and elevated AMP activate AMPK are not [51].

LKB1-AMPK activity antagonizes anabolic processes, such as gluconeogenesis, lipogenesis, protein synthesis, and nucleotide synthesis and stimulates catabolic processes, such as autophagy, glycolysis, citric acid cycle, and fatty acid β-oxidation [57, 59, 60]. In other words, the LKB1-AMPK axis activates ATP generating processes while inhibiting ATP and NADPH consuming processes, which is exacerbated in response to metabolic stress induced by hypoxia or nutrient depletion [61]. Given that previous reviews have dissected LKB1-AMPK signalling pathways [62, 63], this review will focus on the importance of the LKB1-AMPK axis to cell polarity, migration, metabolism, and autophagy.

Genetic screens of *Drosophila melanogaster* oocyte mutants linked LKB1 to epithelial follicle cell polarity. Indeed, *STK11* mutant germline clones developed irregular anterior–posterior axis polarity confirming for the first time that LKB1 is essential to cell polarity [64]. Alternatively, *AMPK*-null mutants displayed irregular cell polarity as apical localized proteins, such as Bazooka and β-catenin, were scattered throughout the cell and accumulated at the basolateral surface [65]. In mammalian cell lines, disrupting AMPK impedes tight junction formation [66] whereas LKB1 localizes to adherens junctions, regulates E-Cadherin expression, and is essential to acinar cell polarity in three-dimensional spheroid models [67].

Given the relationships between cell–cell junctions, cell polarity, and motility [68], in vitro wound healing assays assessed the relationship between cell motility and LKB1 distribution. In non-migratory confluent cells, LKB1 is diffusely spread in the cytoplasm and nucleus whereas motile cells redistribute LKB1 to the leading edge. Immunofluorescence colocalization analysis found that LKB1 complexes with active CDC42, p21-activated kinase, and actin at the leading edge [69]. Although AMPK also accumulates at the leading edge during migration [70], controversy surrounds the role of AMPK on cell motility. One argument is that LKB1-AMPK disrupts the formation of transverse actin arcs at the leading edge weakening stable cell–cell contacts [71], which explains observations suggesting AMPK is essential to ovarian cancer migration [8]. Alternatively, evidence proposing AMPK activation halts migration has been noted using mouse myoblasts [72], smooth muscle cells [73], and esophageal squamous cell carcinoma [74]. However, many of the in vitro evidence supporting the anti-migratory roles of AMPK omit the mechanism of AMPK activation as well as LKB1 activity.

**Fig. 2:**
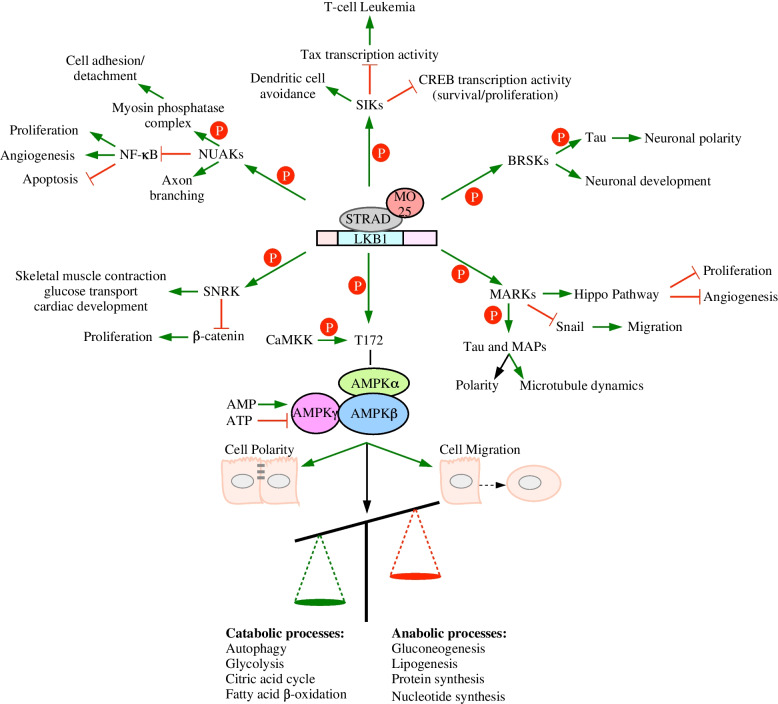
Pathways targeted by the LKB1-STRAD-MO25 complex LKB1-STRAD-MO25 phosphorylate AMPK and AMPK-related kinases (SIKs, BRSKs, MARKs, SNRK, and NUAKs). Both Ca2+/calmodulin-dependent kinase kinase-β (CAMKK2) and LKB1-STRAD-MO25 phosphorylate AMPKɑ on T172. Once phosphorylated AMPKɑ forms an active heterotrimeric complex with AMPKβ and AMPKγ. The active AMPK complex regulates cell polarity, migration, and activates catabolic processes. The AMPK complex is also activated by increasing the AMP:ATP ratio. The LKB1-MARK pathway activates Hippo kinases to impede proliferation and angiogenesis, inhibits Snail to block migration, and regulates polarity and microtubule dynamics by phosphorylating Tau or microtubule-associated proteins (MAPs). The LKB1-BRSK pathway regulates neuronal development and polarity through Tau phosphorylation. The LKB1-SIK pathway antagonizes cyclic AMP response element-binding protein (CREB) and Tax transcription while promoting dendritic cell avoidance. The LKB1-NUAK pathway regulates cell adhesion/detachment and axon branching. NUAK also antagonizes NF-κB activity promoting apoptosis while inhibiting angiogenesis and proliferation. The LKB1-SNRK pathway antagonizes proliferation by inhibiting β-catenin activity and promotes skeletal muscle contraction, glucose transport, and cardiac development

The LKB1-AMPK pathway antagonizes protein synthesis by downregulating mechanistic target of rapamycin (mTOR). This revelation has been proven numerous times via immunoblotting for phosphorylated mTOR and the downstream S6 kinase in experimental models ranging from non-small cell lung cancer cell lines [59] to tissues derived from dual phosphatase and tensin homolog (*PTEN*)-*STK11*-deficient mice [75]. Interestingly, AMPK regulates the transcriptome by phosphorylating and impeding transcription factors that upregulate genes involved with lipogenic and gluconeogenic programs [76, 77]. LKB1-AMPK also regulates carbohydrate, lipid, and protein metabolism through autophagy; a catabolic process that recycles macromolecules and organelles using lysosomal hydrolases [78]. Investigations researching LKB1-dependent autophagy verified that this pathway increases autophagic flux (the rate of lysosomal degradation) via immunoblotting for autophagic markers, electron microscopy of lysosomes and autophagic structures [79], and autophagic flux probes [80]. The LKB1-AMPK axis upregulates autophagic flux through two mechanisms. The first mechanism is through disrupting mTOR function [57]. As the catalytic subunit of mTOR complex 1, mTOR conjugates inhibitory phosphate groups to autophagy-related proteins halting the assembly of autophagic structures and the recruitment of macromolecules to autophagic structures [81]. mTOR also phosphorylates transcription factor EB promoting its cytoplasmic retention through 14–3-3 binding thus downregulating the expression of genes essential for lysosomal biogenesis [82]. The second mechanism of LKB1-AMPK autophagy activation is through activating uncoordinated 51-like autophagy activating kinase 1 [81]. This kinase opposes mTOR activity as it phosphorylates and activates autophagy-related proteins [83]. Regardless of the specific mechanisms underlying autophagy activation, the LKB1-AMPK axis facilitates the breakdown of carbohydrates, lipids, and proteins to their basic building blocks (Fig. 2).

### Discovering AMPK-related kinases (ARKs)

Searching for LKB1 substrates, Lizcano et al., 2004, aligned T-loop sequences of kinases that share both consensus motifs and a conserved catalytic threonine with AMPK [30]. These kinases were classified as AMPK-related kinases (ARKs) and included microtubule affinity-regulated kinase 1–4 (MARK1-4), brain-specific kinase 1 and 2 (BRSK1/2), salt inducible kinase 1–3 (SIK1-3), and NUAK family kinase 1 and 2 (NUAK1/2). Similar experiments later revealed that sucrose non-fermenting-related kinase (SNRK) was phosphorylated within the T-loop by LKB1 [84]. Therefore, the ARKs implicated in LKB1 signalling include MARK1-4, BRSK1/2, SIK1-3, NUAK1/2, and SNRK.

ARKs share structural similarities with AMPK, such as a homologous catalytic threonine in the N-terminal T-loop serine-threonine kinase domain targeted by LKB1 [30]; however, most also possess a ubiquitin-associated (UBA) domain and variable C-terminal spacer sequences [85]. Apart from NUAK1/2, ARKs are the only kinases in the human genome that contain a UBA domain [50]. Unexpectantly, these domains do not bind ubiquitin. Instead, a small-angle X-ray scattering analysis revealed that prior to activation ARKs undergo a conformation rearrangement that brings the UBA domain and kinase domain in close proximity suggesting that these domains are important for activation and targeting downstream substrates [85]. Another major difference between AMPK and ARKs is evident in the presence of AMPK activators. AMPK agonists do not alter the activities of ARKs suggesting that their regulation differs from AMPK [86]. For instance, NUAK1 is activated by cAMP-dependent Protein Kinase C (PKC)-mediated increases in [Ca^2+^] but is not influenced by Calcium/calmodulin-dependent kinase kinase-β (CAMKK2) or transforming growth factor beta-activated kinase 1 (TAK1) [87], which phosphorylate AMPK. CAMKK2 has also been excluded from BRSK1/2 activation [88] whereas the role of TAK1 remains elusive. Finally, CAMKK2 does not activate the ARKs expressed in HeLa cells (SIK1-3, NUAK2, and MARK1-4) [89]. Therefore, upstream kinases and regulatory proteins vary for different ARK family members, which is extensively reviewed elsewhere [50].

### The functions of LKB1-AMPK-related kinases (ARKs)

siRNA mini screens of kinases downstream of LKB1 indicated that LKB1 controls the activity of hippo kinases through MARK1/3/4 [90]. Given that the hippo pathway is linked to cell proliferation and apoptosis, the LKB1-MARK-hippo kinase axis may play a role in the tumour suppressing functions of LKB1. Indeed, *MARK3* overexpression decreases angiogenesis and proliferation in high grade ovarian cancer [47]. The LKB1-MARK pathway also regulates tumour metastasis as cell migration and invasion are induced by LKB1, MARK1 or MARK4 inactivation. Further analysis revealed that inactivation of the LKB1-MARK pathway increased expression of an epithelial-mesenchymal transition-transcription factor called snail [91, 92]. Each MARK family member has two phosphorylation sites in the activation loop where one is an inhibitory regulation site, and the other is targeted by LKB1 to enhance activity [93]. Once activated, MARK1-4 are master kinases of cell polarity and control cellular organization, differentiation, and cell division [94]. MARKs regulate cell polarity through phosphorylating microtubule-associated proteins, such as tau, Microtubule-associated Protein (MAP)2, and MAP4 in the KXGS motif of the microtubule-binding domain. Cytoskeleton stability and cell polarity depend on the LKB1-MARK axis because MARK disrupts the microtubule binding domains of several MAPs to increase microtubule dynamics [95, 96]. Although MARK1-4 localize to the cytoplasm to destabilize microtubules, MARK4 is also found in the nucleus and associates with centrosomes where it may regulate proliferation [96].

The generation of a cortical neuron specific *STK11*-knockout mouse revealed that LKB1 is essential to neuronal polarization. Although overall brain cortex size of transgenic mice were normal, the cortical walls were thinner and the ventricles were enlarged compared to wild-type mice [97]. Given that this phenotype was also observed in *BRSK1/2* knockout mice and BRSK1/2 are LKB1 substrates, it was postulated that the LKB1-BRSK axis controls neuronal polarity [98]. Indeed, BRSK1/2 localizes to the hippocampus and cerebellum in mouse brains [97] and cortical neurons fail to develop axonal and dendritic processes upon *BRSK1/2* or *STK11* inactivation [99]. Like MARKs, BRSK proteins phosphorylate MAPs such as tau to increase microtubule dynamics, which regulates cell polarity [100].

*SIK1-3* are predominately expressed in neural tissues and regulate dendritic cell avoidance to prevent clumping and disorganization of dendrites [101]. It is hypothesized that these kinases facilitate tumour suppressing functions of LKB1 because both LKB1 and SIK are essential to decrease cyclic AMP response element-binding protein (CREB)-dependent transcription [102, 103], which is often constitutively active or overexpressed in tumour specimens because CREB upregulates processes favourable for survival and proliferation. Furthermore, the LKB1-SIK axis inhibits Tax, a transcription factor responsible for upregulating human T-cell leukemia virus type 1 to induce T-cell leukemia [104]. Using genetically engineered mouse models of pancreatic cancer, Patra et al., 2018, first illustrated that suppressing SIK activity in vivo lead to tumour formation [105]. Murray et al., 2019, verified these finding by generating lentiviral Cre recombinase vectors encoding single guide RNAs against SIK1-3 to target *KRAS*^*G12D*^ driven lung adenocarcinoma in mice. Indeed, lung tumour proliferation and stem cell like properties were induced by *SIK1* and *SIK3* knockout. Given that the similarities of histological features and gene expression changes in lung tumours obtained from *STK11* and *SIK* knockout mice, SIKs mediate tumour suppressing functions of LKB1 [106].

In orthotopic mouse models of ovarian cancer, both LKB1 and NUAK are essential for invasion and metastasis [107]. Transcriptomic analysis of high grade serous ovarian cancer indicated that LKB1-NUAK1 loss upregulated Nuclear Factor Kappa B (NF-κB) signalling [108]. NF-κB is a transcription factor that links cancer and immunogenic processes and is often upregulated in tumours to promote proliferation and angiogenesis while suppressing apoptosis. Yet, in ovarian cancer, inhibiting NF-κB promotes tumour growth suggesting a potential mechanism to explain the pro-tumourigenic properties of LKB1 signalling in ovarian cancer [109]. Little is known regarding the LKB1-NUAK pathway in addition to tumourigenesis. What is known is the LKB1-NUAK1 pathway regulates cell adhesion and detachment, and NUAK1 stimulates cell detachment through phosphorylating myosin phosphatase complexes, thereby suppressing phosphatase activity via 14–3-3 binding [110]. This information also links LKB1 to neural development, as disrupting LKB1-NUAK signalling decreased axon branching by increasing mitochondrial mobility [111].

A tissue distribution analysis suggests that *SNRK* is primarily expressed in the brain, adipose tissue, and testis [84]. Since its identification as a LKB1 substrate, *SNRK* knockout mice have highlighted its role in skeletal muscle contraction and glucose transport [112], cardiac development [113], and tumourigenesis. A gene array of colon cancer determined that *SNRK* expression was inversely correlated with the expression of proliferative genes. In fact, *SNRK* overexpression decreased colon cancer proliferation, which was attributed to decreasing β-catenin protein levels, nuclear translocation, and the expression of genes targeted by β-catenin that drive cell cycling [114] (Fig. 2).

## Regulating LKB1-STRAD-MO25 activity

Given that LKB1 localizes to the cytoplasm [24], nucleus [115], and cell membranes [116] to modulate numerous biochemical functions, as well as the mitochondria during apoptosis [117], a diverse set of processes regulate the localization and activity of the heterotrimeric complex. *STK11* inactivating mutations directly antagonize kinase activity and protein–protein interactions. Although other mutations to *STRAD* or *MO25* are less known and are not screened, they would also impact LKB1 activity. Furthermore, allosteric mechanisms, binding to accessory proteins and membranes, and post-translational modifications regulate LKB1 kinase activation, cellular distribution, stability, and/or substrate selection [31, 32, 116, 118, 41].

### Regulating LKB1 function through protein–protein interactions

Despite not influencing LKB1 kinase activity or substrate specificity, (HSP)90 and cell division cycle 37 (CDC37) binding protects LKB1 from proteasome-dependent degradation [119]. Furthermore, co-immunoprecipitation assays demonstrated that LKB1 T336 is essential for 14–3-3 binding [120] but non-phosphorylated LKB1 may associate with 14–3-3 via hydrophobic interactions [121]. 14–3-3 binding does not change the cellular distribution of LKB1 but phosphorylation of downstream substrates is blocked [120]. However, despite 14–3-3 blocking LKB1-dependent phosphorylation, 14–3-3-LKB1 complexes may still regulate the localization and activity of some downstream kinases, such as SIK3 [122].

Given that Fyn tyrosine kinase was linked to LKB1-dependent processes, such as fatty acid oxidation, energy expenditure, and AMPK activity, it was suspected of being an LKB1 interacting partner [123]. Analyzing the LKB1 primary amino acid sequence revealed a proline-rich motif (321PIPPSP326), which is a common binding site for the SH3 domain of Fyn kinases [124]. As suspected, Fyn and LKB1 co-precipitate and mutations in either the proline-rich motif or SH3 domain decreased co-precipitation [124]. After Fyn binds LKB1, the proportion of phosphorylated tyrosine increased, which verified that LKB1 was phosphorylated by Fyn. A Phosphosite Detector identified potential tyrosine phosphorylation acceptor sites on LKB1 as mutating Y261/365 of *STK11* significantly decreased tyrosine phosphorylation [125]. Fyn kinases disrupt LKB1 catalytic activity as overexpression and knockdown of *Fyn*antagonised and induced AMPK phosphorylation, respectively [126],. In support of this, inactivating Fyn with inhibitors or mutations redistributed LKB1 to the cytoplasm and increased AMPK activity [125].

**Fig. 3:**
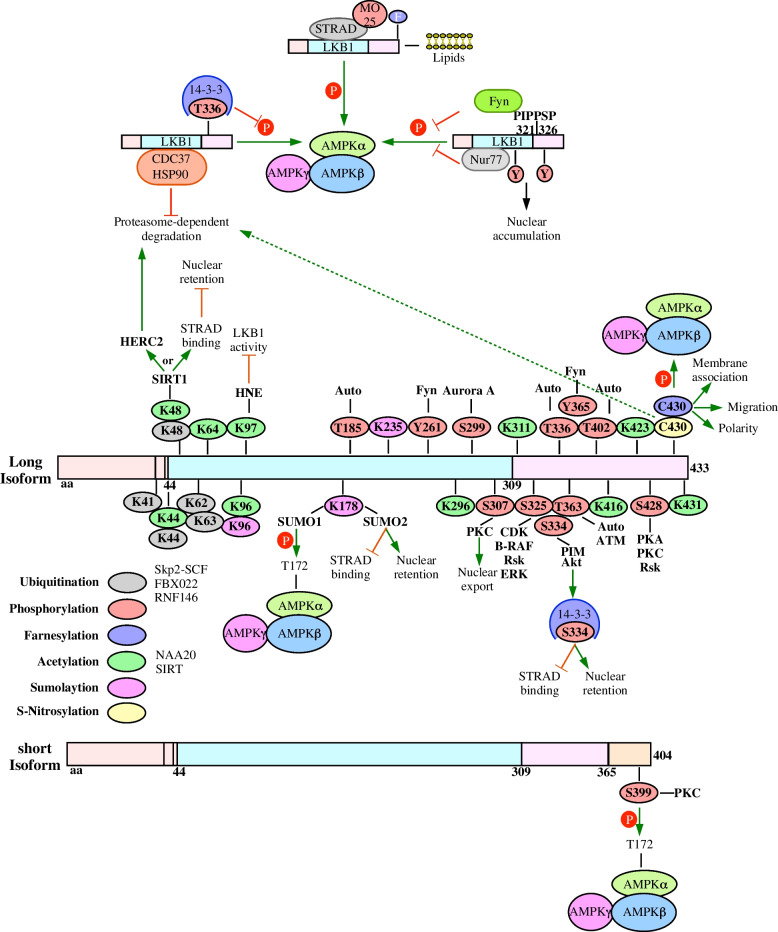
Regulating the functional status of LKB1 LKB1 activity is regulated through protein-protein interactions, lipid binding, and post-translational modifications. Heat-shock protein (HSP) 90 and cell division cycle 37 (CDC37) increase LKB1 activity (measured by AMPK activation) via stabilizing LKB1 and blocking its proteasome-dependent degradation. However, LKB1 activity is disrupted by 14-3-3 binding to phosphorylated T336, Fyn binding to PIPPSP motifs and phosphorylating tyrosine residues (Y), and Nur77 binding, which all promote LKB1 nuclear retention. Several post-translational modifications including ubiquitination, phosphorylation, farnesylation, acetylation, sumolation, S-Nitrosylation, and 4-hydroxy-trans-2-nonenal (HNE) adducts may enhance or antagonize LKB1 activity. Ubiquitination of K41, K44, K48, K62, and K63 are mediated by Skp2-SCF, FBXO22, and RNF146. There are several sites of autophosphorylation on LKB1 including T185, T336, T363, and T402. Upstream kinases including proviral integration site for MuLV (PIM), protein kinase B (Akt), cyclin-dependent kinases (CDKs), v-Raf murine sarcoma viral oncogene homolog B1 (B-RAF), Rsk, extracellular signal-regulated kinase (ERK), Aurora A, and Fyn inhibit LKB1 through Y261, Y365, S299, S325, and S334 phosphorylation whereas protein kinase A (PKA), protein kinase C (PKC), Rsk, and ataxia telangiectasia mutated (ATM)-dependent phosphorylation at T363, S307, S399, and S428 enhance LKB1 activity. Farnesylation at C430 increases LKB1 activity, promotes LKB1-membrane association, and is key for LKB1 regulating migration and polarity. Acetylation of K44, K48, K64, K96, K97, K296, K311, K416, K423, and K431 is regulated through a balance of activity by N-ɑ-acetyltransferase 20 (NAA20) acetylases and sirtuins (SIRTs) deacetylases. SIRT activity has been associated with HECT and RLD domain containing E3 ubiquitin ligase 2 (HERC2) ubiquitinating LKB1 prior to its degradation via proteasomes. Alternatively, SIRTs may enhance LKB1 activity by increasing its affinity for STRAD. Sumolaytion of K96, K178, and K235 is mediated by SUMO1 and SUMO2. SUMO1 enhances LKB1-dependent AMPK phosphorylation whereas SUMO2 disrupts STRAD binding, which increases LKB1 nuclear retention. S-Nitrosylation of C430 dampens LKB1 activity by promoting its degradation via proteasomes. Another antagonist of LKB1 activity is the formation of HNE adducts on K97

As previously discussed, STRAD and MO25 are non-covalent LKB1 binding partners that mediate allosteric LKB1 kinase activation. Examining the LKB1-STRAD-MO25 crystal structure suggests that STRAD alters LKB1 positioning to the active confirmation and MO25 stabilizes LKB1-STRAD complexes and the kinase domains activation loop. However, 14–3-3 and Fyn disrupt LKB1 function by either blocking substrate access to the kinase domain or altering the cellular distribution of LKB1, respectively. In addition to Fyn, nuclear proteins, such as Nur77, promote the nuclear retention of LKB1 to reduce its kinase activity [115]. Furthermore, a C-terminal lipid binding domain and farnesylation site mediates LKB1 binding to membrane phosphatic acids, which may be necessary to fully activate its kinase activity [116]. Together, LKB1 binding to phosphatic acids, MO25, and STRAD increases active site accessibility. In summary, LKB1 binding proteins can stabilize LKB1 (HSP90 and CDC37), enhance enzymatic activity and nucleocytoplasmic shuttling (STRAD and MO25) or antagonize LKB1 function (14–3-3 and Fyn) (Fig. 3).

### Regulating LKB1 function through post-translational modifications

Despite binding to similar accessory proteins and lipids, the cellular localization, kinase activity, and protein interactions of LKB1 can be variable [127]. Over the years, numerous post-translational modification sites of LKB1 have been recognized and may account for this variability. Mutating these sequences alters protein localization, conformation, and/or function, as well as creates novel interacting sites for substrates and accessory proteins [128, 129].

Phosphorylation is the most common post-translational modification of LKB1 residues, which is mediated by upstream kinases, LKB1 binding proteins or other LKB1 residues. For instance, STRAD binding induces LKB1 autophosphorylation at T185 and T402 [31]. Another potential, yet controversial, LKB1 autophosphorylation site is T189. LKB1 autophosphorylation was inhibited by mutating T189 to A189, which was initially interpreted as evidence of T189 autophosphorylation and that T189 regulates the phosphorylation of other residues [117]. However, peptide mapping was not performed to verify these results and two tryptic peptide maps generated since do not support T189 as a site for autophosphorylation [31, 130]. Instead, it is plausible that mutating T189 suppressed LKB1 catalytic activity thus suppressing autophosphorylation of all residues [130]. Sapkota et al., 2002, identified four LKB1 autophosphorylation sites on S31, S325, T336, and T363. However, further analysis suggests that only T336 and T363 are autophosphorylated [130]. Although autophosphorylation had no effect on LKB1 localization, substrate specificity or kinase activity in vitro, the growth suppressing functions of LKB1 were dependent on T336 and T363 phosphorylation [130]. Therefore, further experimentation is required to discern the effect of LKB1 autophosphorylation on its activity.

Kinase assays revealed that LKB1 phosphorylation is also mediated by proviral integration site for MuLV (PIM) kinases [131], cAMP-dependent protein kinase A (PKA) [132], Akt [133], cAMP-dependent protein kinase C (PKC) [12, 134], Rsk [13], B-RAF [135], extracellular-regulated kinase (ERK) [136], Aurora A kinase [128], cyclin-dependent kinases (CDKs) [137], ataxia telangiectasia mutated (ATM) kinase [138], and Fyn kinase [125]. The serine, threonine or tyrosine residues phosphorylated by upstream kinases dictate LKB1 activity. Kinases that are known to inhibit LKB1 activity are PIM, Akt, CDKs, B-RAF, Rsk, ERK, Aurora A, and Fyn. All three PIM kinases (PIM1-3) and Akt antagonize LKB1-dependent AMPK activation by phosphorylating LKB1 on S334 [131]. Phosphorylation of LKB1 on S334 promotes LKB1 binding to kinase suppressing 14–3-3 proteins, decreases STRAD binding, and increases LKB1 nuclear localization [133]. LKB1 kinase activity is also antagonized after the Cyclin D1 complex, including CDK4/6, or B-RAF and downstream kinases, such as Rsk and ERK, phosphorylate LKB1 at S325 [135, 137]. As previously discussed, LKB1 activation is disrupted by Fyn-dependent phosphorylation of Y261/365 [125]. In H1299 non-small cell lung cancer cells, Aurora A kinase S299-dependent phosphorylation of LKB1 disrupts LKB1-AMPK signalling and augments tumour cell invasion [128]. Alternatively, kinases that enhance LKB1 activity include PKA, PKC, Rsk, and ATM. In response to DNA damage, ATM phosphorylates LKB1 at T363 to activate AMPK and mediate DNA repair via homologous recombination [139]. Furthermore, LKB1-dependent growth arrest is dependent on PKA or Rsk-mediated phosphorylation of murine LKB1 on S431, which is equivalent to human S428 [140]. Indeed, PKA or PKCζ-dependent phosphorylation of human LKB1 on S428 activates AMPK activity [12, 141]. Also, PKCζ promotes the nuclear export of LKB1 after it phosphorylates LKB1 on S307 [12, 142]. In conclusion, depending on the residue phosphorylated by upstream kinases, LKB1 activity may increase (T363, S307, and S428) or decrease (Y261, Y365, S299, S325, and S334).

Although phosphorylation is the most common form of LKB1 post-translation modification, LKB1 is also susceptible to ubiquitination, ribosylation, sumoylation, acetylation, neddylation, and farnesylation. Ubiquitination assays found that ubiquitin ligases, such as Skp2-SCF [143], FBXO22 [129], and RNF146 [144], polyubiquitinate LKB1 on five N-terminal lysine residues (K41, K44, K48, K62, and K63) [143]. Since K48-linked polyubiquitination may trigger LKB1 degradation via proteasomes and K63-linked polyubiquitination regulates protein function, cellular localization, and interactions, knowing the specific type of polyubiquitinated chains is important [145–147]. Additionally, identifying specific lysine residues ubiquitinated by each ubiquitin ligase is important because K63-linked ubiquitin chains have been demonstrated to either activate or antagonize LKB1 activity, which are both implicated in pathogenesis. For instance, oncogenic Ras promotes hepatocellular carcinoma survival using Skp2-SCF ubiquitin ligases to activate LKB1 and promote LKB1-STRAD-MO25 complex formation [143]. Alternatively, non-small cell lung cancer develops when LKB1 signalling is inhibited by FBXO22 [129], and cardiac hypertrophy occurs after LKB1 signalling is antagonized by RNF146 [148]. RNF146 polyubiquitination was suppressed by mutations to tankyrase binding motifs within *STK11* (STK11-R42/G47/R86/G91A). Given that RNF146 only co-precipitates with ribosylated LKB1, LKB1 ribosylation precedes RNF146-dependent ubiquitination [144].

Sumolyation is the covalent attachment of small ubiquitin-related modifier (SUMO) 1–5 proteins to lysine residues. LKB1 K96, K178, and K235 residues are within consensus sumolation sites (branched chain amino acid, lysine, non-specific amino acid, glutamic acid) [149]. However, the possibility that LKB1 may be sumoylated on other sites cannot be negated because approximately 40% of known sumoylation sites occur on lysine residues outside consensus motifs [150]. Given that SUMO-specific protease 1 knockdown increased SUMO levels following LKB1 pulldown, sumoylation of LKB1 was confirmed. Ritho et al., 2015, observed that metabolic stress increased SUMO1 modification of lysine K178 and that this SUMO-interacting motif is essential to LKB1-AMPK interaction as well as AMPK activation [151]. Furthermore, after K48 is conjugated to an acetyl group in human hepatoma cells, K178 is sumoylated by SUMO2. Unlike SUMO1 sumoylation, SUMO2 sumoylation disrupts LKB1-STRAD binding and nucleocytoplasmic shuttling [149].

Like ubiquitination and sumoylation, neddylation relies on E1-activating, E2-conjugating, and E3-ligasing enzymes to covalently modify proteins. However, unlike ubiquitination and sumoylation, there is much unknown about the specific residues modified and the functional consequences of LKB1 neddylation. What is known is that LKB1 neddylation is associated with metabolic reprogramming and poor hepatocellular carcinoma prognosis [152].

As discussed above, acetylation regulates LKB1 sumoylation, kinase activity, and cellular localization [149]. Although few LKB1-targeting acetylases have been discovered, there are several LKB1 lysine residues subject to acetylation (K44, K48, K64 K96, K97, K296, K311, K416, K423, and K431) [153]. N-terminal lysine acetylation mediated by N-ɑ-acetyltransferase 20 blocks LKB1-AMPK signalling [154]. Conversely, sirtuins 1–3 (SIRT1-3)-mediated deacetylation of LKB1 lysine residues may activate or block LKB1 activity [155–157]. For example, SIRT1-dependent deacetylation at K48 promotes the proteosome-mediated degradation of LKB1 through interactions with E3 ubiquitin ligases [155]. Mechanistically, upon acetylation of LKB1, SIRT1 forms a complex with HERC2, HECT and RLD domain containing E3 ubiquitin ligase 2, which conjugates K48-linked ubiquitin chains to LKB1 [158]. Alternatively, overexpressing SIRT1 in HEK293T cells did not result in the proteasome-dependent degradation of LKB1. Instead, SIRT1-mediated deacetylation increased LKB1 cytoplasmic translocation, STRAD association, and activity [153]. Furthermore, a working model outlining why cardiac fibrosis and failure were observed in Rat heart tissues with elevated Pum2 levels revealed that Pum2 mediates SIRT1 mRNA turnover. Loss of SIRT1 in turn increases LKB1 acetylation, which represses the activity of AMPK [159]. Like SIRT1, *sirt2* and *sirt3* knockout mice displayed cardiac hypertrophy because deacetylation at K48 promoted LKB1 phosphorylation and subsequent AMPK activation [160, 161].

LKB1 is farnesylated at cys430 within a conserved CAAX farnesylation site. In several tissues and cell types, disrupting LKB1 farnesylation blunts AMPK activation but the activation of other ARKs is unaffected. Post-translational farnesylation is important to LKB1 activity due to its role in membrane association [116]. Indeed, homozygous *STK11*^*C433S/C433S*^ knockin mice had fewer LKB1-membrane interactions in hepatic cells [140]. LKB1 farnesylation is essential to cell motility because farnesylated LKB1 colocalizes with actin, activates RhoA and Rock, and induces stress fiber formation within lamellipodia at the leading cell edge [162]. Although the LKB1 kinase domain regulates focal adhesion kinase activity, adhesion turnover, and collagen remodelling, the C-terminal farnesylation site mediates cell polarity and migration [163].

Other types of post-translational modifications have been observed in specific cell types subject to an external stimuli or stressor. Lie et al., 2015, demonstrated that LKB1 C430 is modified by S-Nitrosylation in macrophages stimulated with lipopolysaccharide. The clinical significance is that LKB1 Nitrosylation promotes the ubiquitination and proteasome-dependent degradation of LKB1, which may explain why mice stimulated with lipopolysaccharide were susceptible to septic shock and had lower survival rates [164]. In a response to oxidative stress, lipid peroxidation produces 4-Hydroxy-*trans*-2-nonenal (HNE). HNE forms covalent adducts at K97 of LKB1 and although it does not influence its association with accessory proteins, the kinase activity of LKB1 is diminished [127] (Fig. 3).

## The paradoxical nature of targeting LKB1 signalling

Given that mutations to the LKB1-AMPK signalling axis is sufficient for spontaneous tumour formation [165], AMPK agonists are currently under investigations as potential anti-tumourigenic pharmacological candidates. For instance, the biguanide metformin activates AMPK by directly increasing AMP levels, and indirectly through phosphorylating LKB1, which increases LKB1-mediated AMPK activation [12, 166]. Metformin reduces cancer mortality in diabetic patients [167]; increases overall survival in clinical trials [168]; and rescues the immune system from hypoxia induced immunosuppression [169]. As an analog of AMP, AICAR upregulates AMPK activity ultimately decreasing tumour cell invasion and viability [170]. Although pharmacological agents such as Cafestol and β-Ionone do not function as AMPK agonists, they repress tumour progression through activating LKB1-AMPK-dependent autophagy [171, 172]. Activating LKB1-AMPK signalling also has therapeutic potential in non-cancerous pathologies. For instance, the neurotrophic factor, metrn1 relies on LKB1-AMPK-ULK1-dependent autophagy to alleviate diabetic cardiomyopathy symptoms [173].

Despite the therapeutic benefits, evidence that the LKB1-AMPK signalling axis drives pathology is expanding. Briefly, the pro-tumourigenic properties of LKB1-AMPK signalling involve resistance against tumour cell anoikis [174], increasing reactive oxygen species scavenging in tumours by maintaining NADPH levels [175], and autophagy upregulation [137]. In fact, MO25 promotes cisplatin resistance in bladder cancer via LKB1-AMPK-dependent autophagy [176]. In addition to tumourigenesis, LKB1-AMPK mediate fatty acid oxidation in fibroblast-like synoviocytes, which increases proinflammatory phenotypes in rheumatoid arthritis patients [177]. Therefore, in certain contexts, targeting the LKB1-AMPK pathway may have therapeutic merit.

Currently, there are no published pre-clinical nor clinical investigations utilizing LKB1 inhibitors suggesting that its label as a tumour suppressor has delayed the development of LKB1-targeting pharmacological compounds. Despite these delays, there is now a need to synthesize LKB1 inhibitors due to the evidence that LKB1 deficiency enhances the efficacy of other therapeutics. For instance, the selectivity and cytotoxicity of metformin, mitochondria-targeted metformin (mitomet), and erlotinib are improved in LKB1-deficient cells [178–180]. Mechanistically, the LKB1-AMPK axis is critical for cells to maintain reactive oxygen species scavenging, mitochondrial function, and ATP homeostasis, thereby increasing LKB1-deficient tumour cells sensitivity to metabolic stressors [181]. Thus, pharmacological compounds that induce metabolic stress, such as metformin, mitomet, and erlotinib, have therapeutic merit when LKB1 is inactivated suggesting an additive effect if these agents were administered in combination with LKB1 inhibitors. Likewise, PARP inhibitors had greater success at reducing the progression of LKB1-deficient lung cancer taking advantage of DNA repair defects mediated by LKB1 deficiency [182]. *STK11* inactivating mutations also sensitizes lung cancer to ERK inhibitors [183] whereas downregulating LKB1 through miR-17 ~ 92 targeting improved the efficacy of biguanide treatment [184]. Due to LKB1-deficiency enhancing the anti-tumour activity of several compounds in different disease models, future investigations should explore synthesizes novel LKB1 inhibitors.

## Perspectives

### Challenges to assessing STK11 loss-of-function

Immunohistochemical (IHC) staining of formalin-fixed paraffin-embedded (FFPE) tissues detected that *STK11* expression is reduced by 30% in *KRAS*-mutant lung tumours and *STK11* loss is more common in smokers [185]. However, IHC cannot extrapolate *STK11* loss-of-function beyond the study parameters as many *STK11* inactivating mutations will be detected by IHC. Excessive background staining and lack of internal controls may also lead to data misinterpretation [186]. Diagnostic sequencing is utilized to assess *STK11* loss, but epigenetic modifications and mutations with unknown consequences may be missed [187]. Given the role of *STK11* inactivation in pathology and challenges to existing strategies, such as IHC, developing clinical assays to accurately detect *STK11* loss is of concern for researchers and patients alike. This rationale was cited by Chen et al., 2016, which led to the characterization of a sensitive NanoString-based assay to score LKB1 disruption. Indeed, an *STK11* mutation signature generated from thousands of patient samples and cell lines assessed fluorescent probes that hybridize to various *STK11* RNA fixed in FFPE slides with improved accuracy to IHC staining [188]. Despite improvements to the accuracy of strategies assessing *STK11* loss-of-function, these techniques only assess *STK11* levels and *STK11*-specific mutants. Therefore, these tools negate LKB1-associated proteins where a loss-of-function mutation to any of these proteins will disrupt LKB1 activity (Table 1) [29].

**Table 1:** Factors influencing the therapeutic relevancy of LKB1 inhibitors

Factors influencing LKB1 activity	Therapeutic relevancy to LKB1 inhibitors	References
LKB1 mutations	• Mutations to the nuclear localization signal would increase cytoplasmic LKB1 and kinase activity making LKB1 inhibitors therapeutically relevant	[189]
	• Mutations to the kinase domain would produce inactive LKB1 minimizing the efficacy of LKB1 inhibitors	
LKB1 splicing	• Despite binding to STRAD and MO25, short isoforms lack many residues in the C-terminus subject to post-translational modifications making its regulation and activity different from long isoforms	[12, 13]
	• Designing inhibitors to impact the activity of long isoforms may not be beneficial in tissues that preferentially express short LKB1 isoforms	
LKB1 cellular distribution	• Given that LKB1 functions predominately in the cytoplasm, cells with an excess of nuclear LKB1 will be poor candidates for LKB1 inhibitors	[29]
STRAD/MO25 mutations	• These mutations often go undetected but result in a loss of LKB1 activity	[114]
	• LKB1 inhibitors would have little impact if STRAD and MO25 are mutated	
STRAD/MO25 binding	• Inhibiting LKB1 may upregulate LKB1-independent activities of STRAD and MO25	[30, 36]
	• LKB1 inhibition may decrease STRAD and MO25 stability impacting cellular processes modulated by these proteins	
	• Both outcomes have an unknown impact on the risks of LKB1 inhibitors	
LKB1-STRAD-MO25 complexes	• Functional differences between STRADɑ vs STRADβ and MO25ɑ vs MO25β	[38]
	• Due to the alpha and beta isoforms of STRAD and MO25, there are 4 variations of the LKB1-STRAD-MO25 complex where the functional differences remain largely unknown	
	• Inhibiting LKB1 complexes that augment pathology may be beneficial but disrupting other LKB1 complexes may worsen prognosis	
Post-translational modifications	• Alterations to any number of members responsible for LKB1 phosphorylation, ubiquitination, ribosylation, sumoylation, acetylation, neddylation, and farnesylation may impact LKB1 function to augment or disrupt tumourigenesis	[128, 129]
	• Specific alterations to post-translational modifications may increase or decrease the necessity of LKB1 inhibitors	
AMPK expression/activation	• In non-cancerous cells, autophagy regulates homeostasis and protects cells from genetic insults. Disrupting autophagy with LKB1 inhibitors may promote tumourigenesis	[57, 190]
	• Autophagy in tumours is hijacked to promote EMT, anoikis survival, and protects against oxidative and metabolic stressors. Blocking LKB1-AMPK-dependent autophagy may be an effective therapeutic strategy	
AMPK and ARK mutations	• Mutations to downstream kinases may alter LKB1 activity to promote pathology	[163]
	• Depending on the downstream pathways impacted, inhibitors could be either therapeutic or worsen prognosis	
ARK expression and activation	• LKB1 activity varies depending on the downstream ARK	[109]
	• LKB1-NUAK activity may augment tumourigenesis whereas LKB1-MARK and LKB1-SIK have tumour suppressing functions	
	• Blocking LKB1-NUAK activity may be therapeutically relevant, but inhibition of MARKs or SIKs activities may promote tumourigenesis	
Cell/Tissue type	• LKB1 inactivation in lung cancer may induce spontaneous tumour formation whereas ovarian cancer relies on LKB1 activity for spheroid formation, EMT, and invasion	[185, 191]
	• LKB1 inhibitors will be detrimental to pathology in some tissues but beneficial to others	
14–3-3/Fyn binding	• If binding to 14–3-3 and Fyn is disrupted, LKB1 activity will increase	[126, 134]
	• LKB1 inhibitors may be effective for pathologies driven by excessive LKB1 activity due to a lack of 14–3-3 and Fyn-dependent phosphorylation	

### Limitations of linking LKB1 inactivation to disease

Despite linking LKB1 deficiency to pathology, genomic analyses are limited in discerning LKB1 pathways responsible for tumour growth and invasion as the functional consequences of many *STK11* mutants are unknown [192]. An additional drawback is mutations to STRAD, MO25, and other LKB1 binding proteins disrupt LKB1 activity but are negated by studies focusing solely on LKB1 genetics. Furthermore, upstream epigenetic modifications [193] and biological processes downstream of the *STK11* sequence including alternative splicing [21], microRNAs [194], and protein folding [29] may inactivate LKB1 in a manner undetectable using genomic investigative tools alone. Given that *STK11* upstream regulators, downstream effectors, and mechanism of action are not always identifiable using these experimental approaches, the extent of LKB1 deficiency in pathology is likely underreported (Table 1) [106].

### Limitations of experimental procedures utilized to investigate LKB1 activity

While early genomic studies of *LKB1* relied on patient samples, functional assays typically utilize immortalized cell lines with limited physiological relevance. Early studies investigating LKB1 function transfected tagged *STK11* into immortalized human tumour cell lines, such as HeLa, A549, and G361, that do not express endogenous *STK11* [10, 102, 195, 196]. The justification for using these cell lines was largely due to significant functional alterations induced by overexpressing *STK11* and the poor availability of antibodies designed to detect endogenous LKB1. As such, most studies relied on antibodies targeting epitopes on the tag proteins [197–199]. The limitation here is that early LKB1 functional investigations did not monitor endogenous LKB1 activity, splice variants or binding partners, and instead used artificial overexpression systems to draw conclusions on LKB1 biology. These findings must be interpreted with caution because, as reviewed in Prelich 2012, the overexpression of wild-type genes can lead to aberrant phenotypes [200]. Knowing that STRAD or STRAD-MO25 complexes allosterically activate LKB1, it is inappropriate to conclude LKB1 activity based on steady-state protein levels alone.

Given that AMPK was the first LKB1 target identified *in vivo* [51] and the extensive research conducted on the LKB1-AMPK signalling axis, many non-radioactive *in vitr*o kinase assays measure AMPK-dependent phosphorylation to quantify LKB1 activity [12, 13, 151, 201]. The AMPK kinase assay is a two-step process that involves LKB1-dependent AMPK phosphorylation followed by AMPK-dependent phosphorylation of an AMARA peptide or SAMS peptide, which is considered proportional to LKB1-AMPK activity [16, 202]. The drawback of using AMPK phosphorylation to assess LKB1 activity is that other kinases and environmental stimuli facilitate AMPK phosphorylation independent of LKB1 [203, 204]. Buensuceso et al., 2020, observed AMPK phosphorylation in CRISPR/Cas9-dependent *STK11*^*−/−*^ epithelial ovarian cancer cells. In these ovarian cancer cell lines, AMPK phosphorylation was significantly decreased by a CAMKK2 inhibitor, which suggests that AMPK is primarily phosphorylated by CAMKK2 in ovarian cancer [191]. The link between TAK1 and AMPK activation is well documented [205] but interest in the pathway has re-emerged since the TAK1-AMPK pathway was implicated in protection against *Salmonella* Typhimurium invasion [203]. Finally, environmental cell stressors, such as ionizing radiation, have induced AMPK phosphorylation in prostate, lung, and breast cancer cells [204]. Therefore, caution must be warranted when interpreting LKB1 activity based on AMPK-dependent kinase assays alone. Therefore, with a lack of tools and experimental procedures to assess LKB1 activity, pathologies driven by LKB1 activity are likely underreported (Table 1).

### Considerations when designing LKB1 inhibitors

There are several factors contributing to LKB1 stability and activity each impacting the therapeutic relevancy of LKB1 inhibitors. The most important being LKB1-associated protein binding, mutations to components of the LKB1 pathway, and alterations to post-translational modifications. When designing inhibitors of LKB1, researchers must decide between targeting LKB1 kinase activity, STRAD-MO25 binding or specific downstream pathways. Each of these targets presents a unique set of challenges and limitations. Targeting LKB1 kinase activity will ablate all LKB1 activity impacting numerous downstream processes. Although this guarantees LKB1 inactivation, simultaneously disrupting all LKB1 activities may contribute to secondary pathologies and result in unknown consequences to cell biology. If blocking LKB1 enzymatic activity is not viable, targeting STRAD binding may be an alternative strategy.

Instead of focusing on antagonizing LKB1 kinase activity, some investigators are pursuing the development of allosteric modulators of STRAD. Although STRAD allosteric modulators have been shown to directly increase or decrease LKB1 activity, the therapeutic benefit has yet to be explored [206]. However, due to these efforts, it is possible to simultaneously disrupt kinase activity and STRAD binding. However, blocking STRAD-MO25 binding to LKB1 may impact their LKB1-independent activities, protein stability, and cellular localization. Finally, targeting a specific branch of LKB1 activity limits off-target effects but would be both difficult to identify during screening and researchers would need a specific inhibitor for each pathway rather than a single inhibitor. Therefore, prior to designing LKB1 inhibitors, several factors need to be assessed including the therapeutic relevancy of LKB1 inhibitors, the mode of LKB1 inhibition, and potential consequences leading to secondary pathologies (Table 1).

## Concluding remarks

While LKB1 activity is carefully regulated through numerous processes, STRAD and MO25 interactions are paramount to LKB1 cytoplasmic localization and enzymatic activity. What remains largely underreported are the LKB1-independent activities of STRAD and MO25. Both STRAD and MO25 have LKB1-independent roles in cell physiology. For this reason, increasing their availability by blocking their access to LKB1 may distort homeostasis. Alternatively, if STRAD and MO25 are destabilized and degraded in the absence of LKB1, pathways dependent on their function will be negatively impacted. Using this information, it may be beneficial to design inhibitors that block LKB1 kinase activity without impacting STRAD-MO25 binding. Since LKB1 functions as a master kinase regulating many cellular processes, blocking all LKB1 activity may not be desirable. Therefore, more work is necessary to discern the impact of LKB1, STRAD, MO25, and other LKB1 accessory proteins on LKB1 biology and pathology.

Experimental procedures employed to investigate LKB1 biology often fail to accurately assess enzyme activity, LKB1 splicing, *STRAD* and *MO25* mutations, different STRAD and MO25 isoforms, and cellular localization, which has mitigated the role of LKB1 in pathology. In fact, these shortcomings may result in underreporting LKB1 in disease. Given the challenges limiting LKB1 investigations, guidelines to standardize experimental strategies to study LKB1 isoforms, post-translational modifications, and regulation are of upmost importance. To improve experimental strategies to assess LKB1 as a disease biomarker and the therapeutic relevancy of LKB1 inhibitors, this review summarizes what is known about LKB1 activation, downstream targets, and routes to modify enzyme stability/function. This knowledge combined with LKB1-deficiency enhancing the therapeutic efficacy of other anti-tumourigenic compounds should justify the need for designing inhibitors targeting the LKB1 pathway.

## Data Availability

No datasets were generated or analysed during the current study.

## Abbreviations

LKB1: Liver kinase B1
STK11: Serine-threonine Kinase 11
STRAD: STE-20-related kinase adaptor protein
siRNA: Small interfering RNA
MO25: Mouse Protein 25
GST: Glutathione-S Transferase
AMPK: Adenosine Monophosphate-Activated Protein Kinase
mTOR: Mechanistic Target of Rapamycin
PTEN: Phosphatase and Tensin Homolog
ARKs: Adenosine Monophosphate-Activated Protein Kinase-related Kinases
MARK: Microtubule Affinity-regulated Kinase
BRSK: Brain-specific Kinase
SIK: Salt Inducible Kinase
NUAK: NUAK Family Kinase
MELK: Maternal Embryonic Leucine Zipper Kinase
SNRK: Sucrose Non-fermenting-related Kinase
UBA: Ubiquitin-associated
PKC: CAMP-dependent Protein Kinase C
CAMKK2: Calcium/Calmodulin-dependent Kinase Kinase-β
TAK1: Transforming Growth Factor Beta-activated Kinase 1
MAP: Microtubule-associated Protein
CREB: Cyclic AMP Response Element-binding Protein
NF-κB: Nuclear Factor Kappa B
HSP: Heat-shock Protein
CDC37: Cell Division Cycle 37
PIM: Proviral Integration Site for MuLV
PKA: CAMP-dependent Protein Kinase A
ERK: Extracellular-regulated Kinase
CDK: Cyclin-dependent Kinase
ATM: Ataxia Telangiectasia Mutated
SUMO: Small Ubiquitin-related Modifier
SIRT: Sirtuins
HNE: 4-Hydroxy-*trans*-2-nonenal
IHC: Immunohistochemical
FFPE: Formalin-fixed Paraffin-embedded
